# Gas Chromatography Analysis with Olfactometric Detection (GC-O) as a Useful Methodology for Chemical Characterization of Odorous Compounds

**DOI:** 10.3390/s131216759

**Published:** 2013-12-05

**Authors:** Magda Brattoli, Ezia Cisternino, Paolo Rosario Dambruoso, Gianluigi de Gennaro, Pasquale Giungato, Antonio Mazzone, Jolanda Palmisani, Maria Tutino

**Affiliations:** Chemistry Department, University of Bari, via E. Orabona 4, Bari 70126, Italy; E-Mails: magda.brattoli@uniba.it (M.B.); ezia.cisternino@gmail.com (E.C.); paolo.dambruoso@libero.it (P.R.D.); pasquale.giungato@uniba.it (P.G.); antonio.mazzone@uniba.it (A.M.); jolanda.palmisani@uniba.it (J.P.); maria.tutino@uniba.it (M.T.)

**Keywords:** gas chromatography-olfactometry, odors, fragrances, food aromas, sensorial methodology, materials, odor detection port, volatile compounds

## Abstract

The gas chromatography-olfactometry (GC-O) technique couples traditional gas chromatographic analysis with sensory detection in order to study complex mixtures of odorous substances and to identify odor active compounds. The GC-O technique is already widely used for the evaluation of food aromas and its application in environmental fields is increasing, thus moving the odor emission assessment from the solely olfactometric evaluations to the characterization of the volatile components responsible for odor nuisance. The aim of this paper is to describe the state of the art of gas chromatography-olfactometry methodology, considering the different approaches regarding the operational conditions and the different methods for evaluating the olfactometric detection of odor compounds. The potentials of GC-O are described highlighting the improvements in this methodology relative to other conventional approaches used for odor detection, such as sensoristic, sensorial and the traditional gas chromatographic methods. The paper also provides an examination of the different fields of application of the GC-O, principally related to fragrances and food aromas, odor nuisance produced by anthropic activities and odorous compounds emitted by materials and medical applications.

## 1. Introduction

Odors have a direct effect on human behaviors and can significantly affect the quality of life. Evolutionary history has demonstrated the importance of a good sense of smell as it protected our primitive ancestors from predators and helped them to find food. Nowadays, it may not have the same connotation for human survival, but it can most certainly play an important role in human attractions, memories, and emotions. People associate odors with past experiences and, from those experiences, they will involuntarily assess the odor as likable, dislikable or indifferent. These responses are individual and may vary from person to person. Through breathing, humans are continuously testing air quality to obtain relevant information such as potential dangers (e.g., smoke), the presence of food, another individual, and so on. In order to detect an odor, several factors and properties can contribute to generate an olfactory perception [1]:

- *Odor threshold* (OT). This is the minimum concentration at which 50% of a human panel can detect the presence of an odor or odorant without characterizing the stimulus. This is different from the *recognition threshold* which is the concentration that 50% of a human panel is able to detect and describe qualitatively.
- *Physical and chemical properties.* These include the appreciable volatility of a substance at ordinary temperatures (less than 300–400 relative molecular mass) to permeate the air near the sensory area, as well as the slight water-solubility which allows an odor to pass through the mucous layer to the olfactory cells and the lipid-solubility which is necessary since olfactory cilia are composed primarily of lipid material.
- *Intensity.* This is the relative strength of the odor above the recognition threshold. It is logarithmically related to odorant concentration (Stevens' law or the power law) which can be calculated with the following equation [2]:
  (1) I=KlogC
  where I is the intensity, C is the concentration and K is a constant.
- *Hedonic tone.* This is a measure of the pleasantness or unpleasantness of an odor mixture.
- *Quality.* This property identifies an odor and differentiates it from another odor of equal intensity.
- *Molecular structure*. Molecular geometry, in particular, the composition and structure of the functional groups within a molecule, can deeply affect the quality and features of an odor [3].

It should be noted that odors are complex mixtures of many volatile chemicals which are present in different concentrations. These chemicals can interact synergistically or additively in the mixtures according to unpredictable rules [4–6], and are at the basis of the overall sensation of smell.

In the recent years, various research activities have been developed in different scientific disciplines to investigate odors and odor perception with the aim of producing direct applications in industry. Some applications focus on improving the quality of scented products such as perfumes [7] and food products through the use of natural or synthetic odorous compounds. For example, the results of an aroma food analysis can help assess the quality of foods and make them more pleasing and desirable [8]. Others examine the impact of odorous compounds on the quality of human life and the environment. In this case, odors may derive from industrial activities [9–13] (landfills, wastewater treatment plants, refineries, tanneries, and so on) or be emitted from materials that people frequently use [14,15].

The main purpose of odor research is to identify the odor active compounds and to relate them to human perception. Instrumental approaches to the characterization of odorants using Gas Chromatography coupled with Mass Spectrometry (GC/MS) have been widely used to produce lists of the substances present and their concentrations [16,17]. The main limitation of this technique is connected to the complexity of the odor under investigation. Since many volatile chemicals are often present at concentrations lower than the instrumental detection limit and since information about human perception is not provided, a linear correlation between a quantified substance and an olfactory stimulus cannot be made [18]. Notwithstanding the usefulness of GC/MS analyses, the mammalian olfactory system is the most sensitive and inclusive odor detector. Hence, the sensory evaluation of smells by panels of sensory trained evaluators represents a valid approach to odor assessment.

A notable improvement in odor identification consists of coupling GC-MS with olfactometric detection (GC-MS/O). The gas chromatographic separation of an odorous air sample can be useful for identifying specific odorant components. GC-MS/O, thus, allows a better understanding of odorant composition through the identification and quantification of its compounds while offering a partial correlation between the chemical nature of an odorant and its perceived smell [19,20].

This paper will examine the state of the art of the gas chromatography-olfactometry methodology (GC-O), different approaches regarding operational conditions, and different methods for evaluating the olfactometric detection of odor compounds. The potentials of GC-O will be described and its different fields of application such as fragrance and food aromas, industrial and material emissions, and medical applications will be examined.

## 2. GC-O Analysis

### 2.1. GC-O Configuration and Parameters Affecting the Analysis

**Figure 1.**
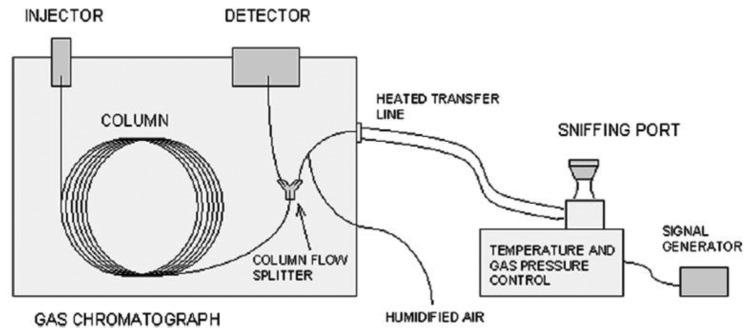
Scheme of the gas chromatograph equipped with an olfactometric detector (reprinted from [22] with permission from Elsevier).

GC-Olfactometry (GC-O) is a valuable method for the selection of odor components from a complex mixture. A properly trained human assessor or a team of them is employed as a detector and can sniff the eluate in order to detect the presence of odor—active compounds via a specifically designed odor port (ODP) connected in parallel to conventional detectors [21] (thermal conductivity, photo-ionization or flame-ionization), as shown in Figure 1.

Each separated compound, eluted by the GC, can be detected by a human assessor (odor present or not), who is able to measure the duration of the odor activity (start to end), to describe the quality of the odor perceived and to quantify its intensity. GC-O in combination with a mass spectrometer (GC-O/MS) not only enables the evaluation of odor compounds, but also their identification with mass spectral information.

In particular, the flow of the eluate is split so that the analytes reach both detectors simultaneously, permitting a comparison of both signals. The retention times of the analytes might differ for the two detectors (typically shorter for the mass spectrometer), due to the fact that the mass spectrometer works under vacuum conditions while the olfactometric detector works under atmospheric pressure conditions. This difficulty can be overcome by installing a restrictor, a narrow bore capillary, before the mass spectrometer to increase the pressure drop between the interface and the flow splitter, as well as through careful selection of the flows of the carrier and auxiliary gases [23].

**Figure 2.**
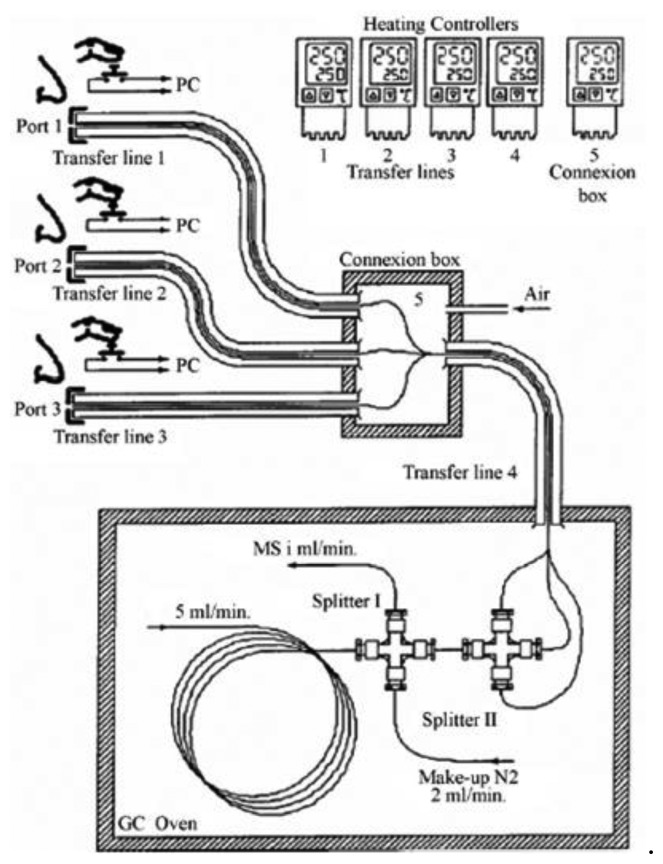
Scheme of the GC/MS-O multi-sniffing system (reprinted from [25] with permission from Elsevier).

The design of all commercially available olfactometric ports is very similar. The eluate reaches the port through an uncoated transfer line (deactivated silica capillaries) and is sniffed in a glass or a PTFE conical port, fitted to the shape of a nose. The transfer line is heated to prevent the condensation of semi volatile analytes on the walls of the capillary. Auxiliary gas (moist air) is added to the eluate to prevent the drying of the assessors' nose mucous membranes, which could cause discomfort especially in longer analyses. The transfer line length can vary, but it must be long enough to ensure a comfortable sitting position and to avoid discomfort due to the vicinity of hot chromatograph components during detection. Each port is also equipped with an electric push-button to generate a signal of 1 V when pressed. If the extract analyzed is sufficiently concentrated, the eluate stream can sometimes be separated into several streams and delivered to more olfactometric ports, for the simultaneous detection by several assessors (Figure 2). This approach can yield more representative results since different sniffers simultaneously smell the sample providing an average value for each analysis [24,25].

A multi-gas chromatography-olfactometry device (eight way gas chromatography olfactometry 8W-GC-O) has recently been developed [26]. This device consists of a chromatograph coupled with a divider that synchronously distributes the volatile component outflow to eight transfer lines which are connected to eight sniffing ports located in separate booths. Flow rates can be set to ensure the best compromise between chromatographic resolution and sufficient effluent at the sniffing ports so that the odorous fractions can be adequately sensed.

There are several factors that determine the quality of the data collected by GC-O. The method used to extract volatile compounds from the samples determines the composition of the extract, and therefore the quality of the eluate available for perception. The set-up of the GC instrument and of the separation conditions affects the quality of chromatography and the response of the human detector. The peak shape affects the perception of odor intensity and the calculation of detection thresholds. Chromatographic behavior of odor substances varies with the compounds and the stationary phases of the GC column. Non polar stationary phases enable the elution of odor-active volatiles at the lowest possible temperature [21]. However, very polar molecules, such as fatty acids, result in poor peak shapes using nonpolar phases. Polar phases demonstrate greater selectivity, although the overall quality of the separation will depend upon the composition of the sample [27].

Moreover, it should be noted that some volatile compounds are labile and will readily decompose in heated injector blocks, forming artifacts; for example, sulfur compounds are particularly susceptible to heat-induced decomposition. The odor character of some compounds depends strongly on their concentration which can become important in the case of poor chromatography or poor chromatographic separation (co-elution). Many key odor compounds often occur at very low concentrations in complex matrixes. Therefore, the identification of odor compounds remains a hard task even with GC-O/MS because some compounds co-elute with other analytes making the correlation between the detected aroma and the correct compound difficult.

Comprehensive two-dimensional gas chromatography (GC × GC) appears to be the most appropriate choice to meet the need for enhanced separation and better sensitivity. This technique is based on the continuous collection of an effluent from a GC column and the periodic reinjection of small portions of the effluent to a second column of different polarity. This novel technique, which associates the resolution power of GC × GC with the selectivity and sensitivity of the human olfactory system, can enable the olfactive analysis of congested chromatographic areas [28–30]. However, GC × GC coupled with ODP is an extremely demanding technique for the operator because peaks are eluted very quickly and the panelist may not have enough time to both recognize odors and provide their descriptors. Respiratory rate can also be critical and an analysis time of more than ten minutes is difficult to achieve.

A direct gas chromatography–olfactometry (D-GC-O) method can be used to perform a representativeness test on the global odor of a sample and select the best conditions for analysis. This recent technique consists of connecting a deactivated capillary column between the GC injector and the sniffing port to avoid chromatographic separation. In this way, the aroma compounds arrive simultaneously at the ODP, where the assessor perceives, evaluates and compares the resulting odor of the starting sample [31,32].

The behavior of the human detector should also be considered during the assessment of the data quality collected when using GC-O. There are very significant differences in olfactory ability between humans; odor thresholds can vary significantly among individuals and some people, with an otherwise normal sense of smell, are unable to detect families of similar smelling compounds (anosmia) [21]. In addition, the olfactory response of an individual is known to vary over time, even during the course of a single day, and with the speed of breathing [33,34]. Sensitivity may also fluctuate due to health status and mood.

In order to obtain reproducible data, potential assessors should be screened for sensitivity, motivation, ability to concentrate, and ability to recall and recognize odor qualities [21]. They should be asked to refrain from smoking and eating/drinking strongly flavored foods for 1 h prior to performing GC-O. They also should not to wear aftershave, perfume or strong deodorants on the day of assessment [21,35]. Moreover, the assessor's comfort and ability to sniff free of distraction should be considered. GC-O instrument should be located in a dedicated laboratory with temperature and pressure control. Finally, a maximum sniff time of 25–30 min is recommended since GC-O sniff duration can impact human detector performance [21].

### 2.2. GC-O Olfactometric Detection Methods

In recent decades, several techniques have been developed to collect and process GC-O data and to estimate the sensory contribution of a single odor active compound, rated as intensity, in order to evaluate the relative influence on the total odor of the sample [36]. These methods can be categorized into three groups: frequency detection methods, dilution to threshold methods, and direct intensity methods.

#### 2.2.1. Frequency Detection Methods

**Figure 3.**
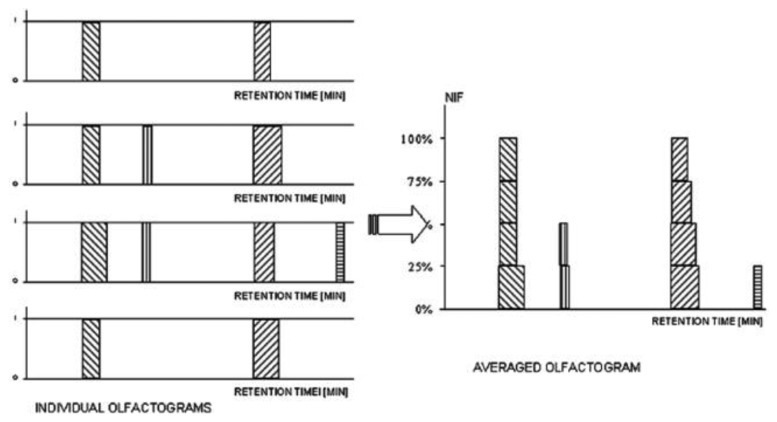
Scheme of an aromagram obtained using detection frequency methods, with four evaluators (reprinted from [22] with permission from Elsevier).

The frequency detection method involves a team of 6–12 people who analyze the same sample in order to provide the percentage of people who sensed the odor compound at a given retention time [37,38]. Each odor can be evaluated using Nasal Impact Frequency (NIF) or Surface of Nasal Impact Frequency (SNIF) values. The NIF value is set at a value of one when each of the evaluators sensed a given odor, and zero when no-one sensed any odor at a given retention time [39]. Therefore, the NIF value corresponds to the peak height of the olfactometric signal. The SNIF values describes the peak areas obtained multiplying the frequency percentage by duration, and enables the production of an aromagram (see Figure 3) [24,38,40]. Simplicity is the main advantage of detection frequency-based methods which do not necessitate qualified evaluators. The methods are repeatable and the results reflect the differences in sensitivity between the evaluators, which can also be related to differences within a given population. Hence, the impact of inattention, specific anosmia, *etc*. on the aromagram is minimized. However, as the detection frequency is related to the intensity of the odor perceived by the assessors, the correlation of the peak height with the real concentration of the odor compound in the sample cannot be obtained. In particular, odorous compounds present in different concentrations, all above the detection threshold, will produce an aromagram with peaks of equal intensity [21,22,41].

#### 2.2.2. Dilution to Threshold Methods

Dilution to threshold methods provide a quantitative description of the odor potential of a given compound based on the ratio between its concentration in the sample and its sensory threshold in air [21]. These methods consist of preparing a dilution series of an extract, usually using twofold, threefold, fivefold or 10-fold dilution levels (R) and then analyzing them with GC-O [42]. The assessors state under which dilution the compound analyzed can still be sensed, and usually describe the type of smell. Odor potency is equivalent to the concept of “aroma values”, “odor values”, “odor units”, “flavor units”, and “odor activity values” (OAV) [21]. OAV is the most commonly used index and represents the ratio of the concentration of a given compound on its sensory detection threshold [43–46].

The most frequently reported dilution methods are “Aroma Extract Dilution Analysis” (AEDA) and “Combined Hedonic Aroma Response Measurement” (CharmAnalysis™) [24,47–57]. AEDA measures the highest sample dilution at which the odor of the analyzed compound is still detectable and reports this as the flavor dilution factor (FD) [36]. If the last dilution under which the analyte is still detectable is equal to *p* (*p* = 0, 1, 2, 3, …), then its dilution factor is R^P^, where R is the dilution level [42]. The overall results obtained with this method are reported in an aromagram presenting the FD value, or its logarithm, against the retention index (RI) [58,59]. CharmAnalysis™ records the duration of odors (start and end) and generates chromatographic peaks. The assessors record the start and the end of each detected odor, hence the aromagram is obtained by plotting the duration of the odor sensation against the dilution value. The peak areas are expressed in dimensionless “Charm” values (C), which are proportional to the amount of the analyte in the sample, and inversely proportional to the sensory detection threshold [22]. Charm value can be calculated with the formula:

(2) $$\text{C}={\text{R}}^{\text{n}-1}$$

where n is the number of coincident odor responses detected at a single retention index and R is the dilution level [60]. CharmAnalysis™ considers both the peak width and the peak shape, thus a short and broad peak may have the same Charm value as a tall and narrow peak that is perceived at a higher dilution. This gives CharmAnalysis™ more discriminating power than AEDA, but it also results in greater variation than AEDA [21].

A drawback of the dilution methods is the length of the total analysis due to the large number of dilutions for each extract and evaluator. Therefore, these methods are normally performed by only one or two evaluators. For example, in a series of 10 dilutions, 30 GC injections are needed, which requires about 2 weeks [61]. Dilution to threshold methods are also criticized for the underlying false assumption that the odor intensity increases in parallel with the concentration for all odor components in a sample [62]. In order to attain a complete analysis of key odorants, recombination models and omission experiments can be performed. In the former, the aroma model system for a specific sample is prepared based on the combination of previously achieved AEDA or CHARM values, and/or OAVs. Odorants showing higher values are used to formulate a recombined model, which is then compared to the real sample for similarity or difference [25]. The omission experiments, on the other hand, deal with the preparation of an aroma model for a specific sample in which one or more odorants are omitted. In this experiment, the panelists are asked to perform duo and triangle tests to compare the reduced model with the complete one and indicate the perceived sensorial differences [25,31,63].

#### 2.2.3. Direct Intensity Methods

The odor intensity and its duration can be measured with direct intensity methods using different kinds of quantitative scales: category scales or unstructured line scales [21]. These methods include a single time-averaged measurement registered after the elution of the analyte (posterior intensity evaluation methods) or a dynamic measurement, where the appearance of an odor, its maximum intensity and decline are registered in a continuous manner (OSME, the Greek word for odor, and Finger span method) [64–66]. In the first case, the assessor assigns an appropriate value, from a previously defined intensity scale, to each detected compound while in the second case, the olfactogram obtained is similar to conventional chromatograms in which the height of the peak corresponds to the maximum odor intensity and the width corresponds to odor duration. Depending on the method, the measurement can be performed in different ways, best results will be obtained if a panel of assessors is used, and the average panel result is treated as one signal. There is a correlation between the logarithm of the odor intensity obtained using the OSME method and the logarithm of the analyte concentration, as described by Steven's Law [29,62]. The intensity is recorded as a function of time by moving the cursor of a variable resistor [25,66]. In the finger span method, the olfactograms are built moving the potentiometer slider using the thumb and the index or middle finger (195 mm) [21,34,67]. The distance between the two is proportional to the intensity of the odor, and the time of sliding corresponds to the duration of the odor in the olfactometric port. Some studies demonstrated that even a completely unprepared team of evaluators is able to repeatedly perform olfactometric measurements [22]. Another device, based on the use of a spring-loaded button can be used to build the aromagram. This device can relate a perceived odor intensity to the physical stimulus of hand pressure, thereby improving the reliability of recorded odor intensity data [41,68]. Using a 3- or 7-point category scale with half values, the data are processed using the modified frequency MF (%), that can determine the most important odorous compounds present in the sample. It can be calculated with the following formula:

(3) $$\text{MF}(\%)=\sqrt{\text{F}(\%)\times \text{I}(\%)}$$

where F (%) is the detection frequency of an aromatic attribute expressed as percentage and I (%) is the average intensity expressed as a percentage of the maximum intensity. Usually, odorous stimuli detected with a MF (%) higher than 50 represent the most important compounds present in each sample [31,69]. This group of methods requires expert evaluators in order to obtain fast, repeatable and generally consistent results even in a single run [21].

### 2.3. Sample Preparation Methods

The choice of an appropriate sample preparation method is crucial in GC-O analyses. The flavor profile is closely related to the isolation procedure which should prevent decomposition of labile compounds, loss of highly volatile compounds and heat-induced artifact formation. Therefore, the selected method should yield a product which is as representative as possible of the sample. According to the properties of the investigated sample, the preparation may include mincing, homogenization, centrifugation, steam distillation (SD), solvent extraction (SE), simultaneous distillation-extraction (SDE), solid phase extraction (SPE), supercritical fluid extraction (SFE), Soxhlet extraction, solvent assisted flavor evaporation (SAFE), microwave-assisted hydrodistillation (MAHD), headspace (HS) techniques, solid-phase microextraction (SPME), matrix solid-phase dispersion (MSPD) and/or methylation, direct thermal desorption (DTD), among others. Conventional solvent extraction and/or distillation methods are widely used to isolate volatile organic compounds present in food, beverages and materials [32,49,51,52,59,70–72]. However, these procedures yield extracts of a sample that do not always reflect the composition of the odor that is perceived by a subject when smelling or eating the sample. In particular, highly volatile compounds that most contribute to the original odor of foods, plants, flowers or materials can be lost during these procedures [73].

Solid phase extraction is carried out by shaking the sample with resin particles or more simply by eluting the sample in SPE columns [45,46]. The SPE resins are usually washed and conditioned with different solvents before sample extraction or clean up [28,74]. Another very popular method is SAFE, which may be applied after SE techniques or be used as an individual extraction method for aqueous samples such as milk, fruit and urine [49]. The technique removes volatile compounds under low temperature and high vacuum conditions. Headspace techniques are used for the sampling of volatiles, using either static or dynamic methods. Sorption traps with porous polymers such as Tenax^®^ TA or Porapak™ Q, and resins such as Lichrolut^®^ EN, are most often used to concentrate collected volatiles [63,66,75,76]. The volatile components are then chemically or thermally desorbed from the trap and analyzed with GC-O [66,75]. Compared to conventional extraction techniques, headspace methods have the benefit of usually not causing the loss of the most volatile compounds and enable chromatographic analysis of these compounds without interferences due to the solvent peak. Headspace volatiles can be also concentrated exposing SPME fibers coated with specific extraction phases such as Carboxen^®^/PDMS or divinylbenzene/carboxen/polydimethylsiloxane [30,31,47,48,50,77–82]. The chemical profile of the collected volatiles depends upon the type, thickness and length of the fiber, as well as on the sampling time and temperature.

Another technique, worthy of note is the direct thermal desorption (DTD), a widely applied solvent-free method [83–87]. For this method, volatile compounds are collected onto adsorption tubes filled with specific resins by using an air pump. The resins are selected according to the type of matrix (Tenax^®^ GR, Carbograph™, Carboxen^®^ GR). Sampled tubes are then thermally desorbed and then analyzed with GC-O [84–87]. Alternately, bags made of inert materials (Nalophan^®^, Tedlar^®^,) are used to collect air samples for analysis with TD-GC-O [83].

## 3. Applications

Because of the its unique features, GC-O is already well established in areas involving fragrance and food aromas and is becoming more and more common in areas related to the environment, medicine and materials. In this section, these principal areas of application are explored and a sampling of results are presented in order to highlight the potentials of this technique.

### 3.1. Food Application

Consumers select and consume food based on three principal properties: flavor, appearance (color) and texture. Flavor is usually divided into the subsets of taste and smell, which are perceived in the mouth and the nose, respectively [60]. It is also defined as the sensation arising from the integration or interplay of signals produced as a consequence of sensing smell, taste, and irritating stimuli from food or beverage [88]. Instead, odor usually refers to the smell of food before it is put into the mouth (nasal perception) while aroma is the retronasal smell of food in the mouth.

The application of GC-MS in this area has marked a real turning point for flavor research. Indeed, the number of known flavors has increased to over 7,000 compounds [89], however, there is no information about odor active components. Gas chromatography in combination with olfactometric techniques (GC-O) can help to detect potent odorants, without knowing their chemical structures, even at very low concentrations and this makes it the only viable method for the selection of aroma-active components from a complex mixture.

GC-O studies on food products focus essentially on three main issues:

(1) The “aroma profile” of various foods and beverages and the dependence between the odor and the chemical composition of the volatile fraction on these products;
(2) The odor changes in food due to processing techniques (fermentation, cooking, the addition of preservatives and flavorings);
(3) The discrimination among a family of foodstuffs (cheese types, coffee).

#### 3.1.1. The Study of “Aroma Profiles”

In recent years, intensive food “aroma profiles” studies have been carried out with the aim of characterizing the substances responsible for odor. In particular, the characterization of the odor-active compounds in different kinds of fruit has been the topic of many papers reported in literature [90–97]. In several studies, Pino *et al.* have investigated the odor-active compounds in fruits such as banana, guava and pineapple [47,98,99]. In these investigations, volatile compounds were extracted from the fresh fruit homogenate headspace using SPME fiber coatings and then introduced in successive sequences into the GC port [100–106]. Moreover, the volatile compounds were examined by isolating the volatile compounds using Simultaneous Distillation-Extraction (SDE) [107–109]. A trained panel of three assessors perceived and evaluated the global odor of the fruits by performing SPME direct gas chromatography (GC-O). The combination of SPME-GC-O and SDE-GC-O detected thirty-one odor-active compounds; eleven of which were reported for the first time as important odorants of banana fruit. Guava fruit volatiles included more than 100 compounds that had been reported in previous studies [110–115], as well as ethyl acetate which had not been previously reported as a major compound. Pineapple fruit volatiles included esters (51), aldehydes (7), alcohols (5), acids (3), terpenes (2), furans (2) and miscellaneous compounds (9) which had all been reported in previous studies, with the exception of methyl 2-methylbutanoate [116–120].

This work reveals two emerging tendencies in GC-O applications: the improvement of repeatability and reliability of the obtained results. These developments were achieved by unifying, simplifying and shortening procedures, especially those involving sample preparation (SPME avoids the use of solvents and the resulting artifacts) and the integration with other extracting techniques, such as SDE. SPME allows the isolation of high and medium volatile compounds whereas SDE can cause their losses and, consequently, an underestimation of their aroma contribution. Hence, the combination of SPME-GC-O and SDE-GC-O can be a way of overcoming this discrepancy in the evaluation of the contributions of volatiles.

Camembert cheese has been studied using aroma extract concentration analysis (AECA) and headspace gas chromatography-olfactometry (HGC-O). This approach revealed the complexity of an odorant matrix in which the most potent odorants are 2,3-butanedione, 3-methylbutanal, methional, 1-octen-3-ol, 1-octen-3-one, phenethyl acetate, 2-undecanone, decalactone, butyric acid and isovaleric acid [121]. The work identified the neutral odorants with the highest OAVs as methanethiol, methional and dimethyl sulphide which contributed to the garlic-like sensory attribute in the odor profile of Camembert. Instead, 1-octen-3-ol and the corresponding ketone were found to be responsible for the mushroom-like sensory attribute while acetic, butyric and capric acid were associated with the acidic sensory attribute.

**Figure 4.**
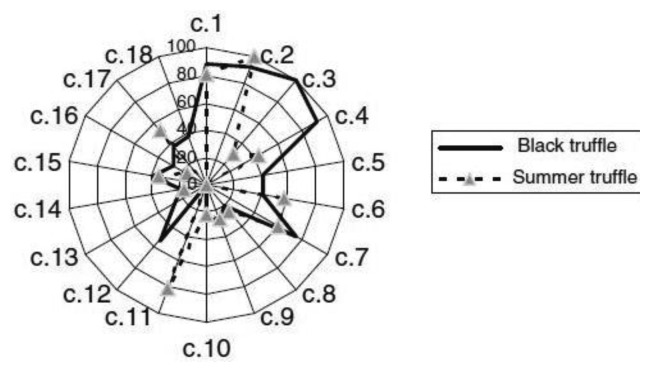
Spider web diagram comparing the GC-O olfactometric profiles (normalized so that the odorant showing maximum MF (%) = 100) obtained from black and summer truffles (reprinted from [122] with permission from Elsevier).

Using two strategies, a recent work [122] has applied GC-O to the characterization of the aroma active compounds in black truffles (*Tuber melanosporum*) and summer truffles (*Tuber aestivum*). The first approach used aroma extract dilution analysis (AEDA), while the second involved a GC-O technique combining measurements of both intensity and modified detection frequency [123–125]. Eighteen different odor zones, with a MF higher than 15%, were detected in the GC-O experiments. The newly identified components were relevant compounds in the aroma composition of the two truffles, particularly in the case of the summer truffles. In fact, 1-hexen-3-one was amongst the five most important aroma compounds. The olfactogram obtained from black truffle was more intense and complex than that of the summer truffle. In addition, the results suggested that there were relevant differences between the aroma profiles of both varieties. In particular, while DMS, DMDS and 3-methyl-1-butanol were among the five most important aroma compounds in both cases, black truffle aroma was rich in 2,3-butanedione and ethyl butyrate while summer truffle aroma contained methional. The comparison between the GC-O profiles of both varieties is shown in Figure 4 where a spider web diagram of the data (normalized so that the maximum = 100%) reveals that the most important differences are related to methional (c.11) and 3-ethylphenol (c.17) which were much richer in the GC-O profile of summer truffle, and 2,3-butanedione (c.3), ethyl butyrate (c.4), ethyl 3-methylbutyrate (c.5) and 3-ethyl-5-methylphenol (c.12), which were particularly important in the aroma profile of black truffle.

Black truffle was particularly rich in phenols (3-ethyl-5-methylphenol, 5-methyl-2-propylphenol, given as mass of 3-propylphenol and 3-ethylphenol, respectively) and in β-phenylethanol, while the emissions of summer truffle was mostly a product of β-phenylethanol, DMS and 3-ethylphenol.

The characterization of aroma-impact compounds in yerba mate (YM) using GC-O and GC-MS was carried out to clarify consumer preferences of major commercial brands of YM sold in Uruguay and its neighboring countries (Argentina, Brazil and Paraguay) [69,126]. For this purpose, all the samples studied were extracted with an identical extraction system and experiments were performed with a system that represented an “artificial mouth”. Approximately 50 odorants were detected during the GC-O experiments but, for simplicity, those not reaching a maximum GC-O modified detection frequency (MF) of 50%, were considered as noise. Sixteen odor-active compounds presenting MF ≥ 50 [127] were detected (Table 1).

Intensive studies have been carried out using gas chromatography with olfactometric detection (GC-O) to evaluate the sensory activity of the individual odorous components of different alcoholic beverages. In these cases, sample preparation represents a critical step since exhaustive extraction methods, such as solvent extraction and distillation, are time consuming, involve many steps and do not always reflect the composition of the odor reaching the receptors during their actual consumption. Moreover, during the concentration step, oxidation of volatiles may occur without the use of antioxidants. Isolation methods are the most used, as static and dynamic headspace with purge and trap (on Tenax^®^ TA or Porapak™ Q, as well as resins, such as Lichrolut^®^ EN), followed by thermal desorption or solvent elution, or Solid Phase Microextraction (SPME) [22]. In alcoholic beverages, GC-O is most commonly used to investigate odor compounds in order to reconstruct alcoholic beverage odors, check the quality of the raw materials used in the production processes and identify the compounds responsible for the aftertaste [128].

#### 3.1.2. The Study of Odor Changes in Food after Technological Treatments

Another interesting area of research concerns the generation of different aroma compounds in foods as the result of technological treatment and the consequent changes of sensory characteristics. In one study, Feng *et al.* set out to evaluate the crucial impact of fermentation on soy sauce aroma [77]. An aroma extract obtained with SPME of a harvested koji sample was subject to GC-O analysis. The results detected 2-phenylpropenal and di-*epi*-α-cedrene for the first time and concurred with previous findings that had noted the presence of volatile [129–131] or aromatic compounds [132–134] as well as.

GC-O combined with the aroma extract dilution analysis (AEDA) approach [59,135] was used to determine key odorants after 1 and 5 days of fermentation and the subsequent frying of soy tempeh. The volatile compounds isolated from the tempeh represented different chemical classes, mainly aldehydes and ketones, hydrocarbons, mono and sesquiterpenes, sulfur containing compounds, nitrogen containing compounds, alcohols, and furans.

A recent study has investigated a new alternative antioxidant in meat originating from a natural plant source [48]. In this work, Kim *et al.* studied the effects of the addition of two commercial rosemary extracts (RE), namely RMD (oil-soluble type) and RMP (water-soluble type), on potent odorants in cooked beef extracts (BE) using solid-phase-micro-extraction-gas chromatography-olfactometry (SPME-GC-O) [136]. The sensory evaluation results indicated that the addition of RE influenced the odor character of cooked BE and thus analysis using SPME-GC-O coupled with AEDA was subsequently conducted to identify the odorants responsible for this trend. A combined total of 57 odorants including 10 unknowns were detected in the treatments. Kim *et al.* concluded that two effects, namely odor-supplementation and odor-suppression, were triggered by the addition of RE. The odor-supplementation consisted in an addition of sweet and floral notes to BE; these odorants were mainly esters, terpenes and phenolic compounds which had originated from the RE, and most notably from the RMD. This effect was thought to have either caused pleasant flavors or masked certain off-flavors in the cooked beef. Instead, the odor suppression effect was mainly observed for the odorants generated from lipid oxidation or the Maillard reaction [137–143]. The study highlighted that CG-O could be a useful tool to monitor and optimize some treatments.

**Figure 5.**
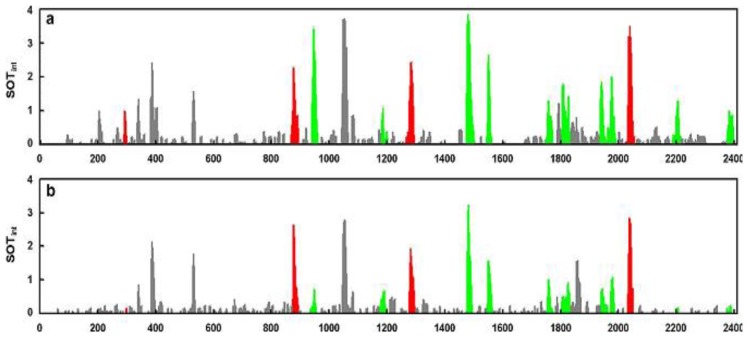
GC-MS/8O aromagrams of cooked hams without (**a**) and with (**b**) nitrite expressed in mean intensities of perception, each calculated from 16 individual sniffing sessions (one type of ham × 8 sniffers × 2 repeats). The breakdown of the signal into three classes of chemical origin shows the odorant zones originating from: lipid oxidation (in green), sulfur compound degradation (in red) and unspecified origins (in grey). (reprinted from [75] with permission from Elsevier).

Another interesting study addressing the use of GC-O in evaluating the effect of technological treatments on odor characteristics was carried out on cooked pork products [75]. This study set out to obtain new knowledge about how nitrite helps to develop aroma in cooked pork products and to describe the reaction mechanisms involved [144–151]. For this purpose, two complementary GC-MS/O instruments were used to identify odorant compounds (GC-MS/8O and GC × GC-MS/O) [26,152]. Using GC-MS/8O, the authors identified 22 odorant zones that subsequently were explored in detail with GC × GC-MS/O for a reliable identification of odorant compounds in the nitrited or nitrite-free cooked ham headspaces. Among the detected compounds, several oxidation products were identified. Indeed, meat fatty acid oxidation explained the presence of the numerous saturated or unsaturated aldehydes with 6–10 atoms of carbon, ketones and alcohols [153,154]. In addition, the authors revealed sulfur compounds resulting from the breakdown of sulfur-containing precursors during cooking [155]. A comparison of the aromagrams of nitrite-free (Figure 5a) and nitrite-cured cooked hams (Figure 5b) is reported, showing that the odor intensity of most of the olfactory peaks was weakened by the added nitrite. Moreover, the authors concluded that an analysis of the differences observed indicates that the zones of the aromagrams most significantly changed (*p* < 0.01) by nitrite correspond to fatty acid oxidation products (green peaks in Figure 5).

In conclusion, the authors demonstrated that the addition of nitrited salt in ham production is not directly involved in the production of the odorous substances that give nitrite cured pork products their specific aroma. Indeed, the absence of nitrite simply promotes the oxidation of fatty acids and, in particular, the production of aldehydes which will mask the odor of the sulfur-containing compounds responsible for the aromatic note typical of nitrite-cured pork products. Thus, the odor of nitrite-cured pork products is merely the outcome of a balanced perception of certain sulfur-containing compounds and fatty acid oxidation products, important among which are aldehydes.

GC-O analysis, based on a detection frequency method has also been used to describe the aroma attributes of beef like flavors (BFs). This has been carried out to determine aroma-active compounds and identify volatile compounds in oxidized tallow samples [156] as well as to clarify the influence of enzymatic hydrolysis-mild thermal oxidation on the odor produced to obtain oxidized tallow. GC-MS profiles of oxidized tallow were analyzed together with quantitative descriptive sensory data and GC-O responses of BFs to understand which compounds had significant effects on aroma-active compounds and sensory attributes of BFs. Through the analyses, the characteristic flavor precursors from enzymatic hydrolysis thermal oxidation tallow were identified and the main differences between enzymatic hydrolysis-thermal oxidation tallow and simple thermal oxidation tallow were elucidated. Compounds with detection frequencies greater than 50% were considered characteristic flavor components; a total of 34 aroma-active compounds were identified, mainly consisting of heterocyclic sulphur or nitrogen compounds and aldehydes. Among these compounds, the most potent odorants were acetic acid, nonanal, 3-(methylthio)propionaldehyde, 2-methyl-3-furanthiol and bis(2-methyl-3-furyl)disulphide. The study also identified bis(2-methyl-3-furyl)disulphide and 2-methyl-3-furanthiol, the latter which is responsible for beef like aroma had also been detected in previous studies [154,157–164].

A similar study was carried out for the evaluation of the changes in the aroma characteristics of mutton process flavors (MPFs) prepared from sheep bone protein hydrolysates (SBPHs) with different DHs (degrees of hydrolysis) using descriptive sensory analysis (DSA) and analyzing the corresponding volatile odor-active compounds with GC-MS/O [165]. The results showed 58 odor-active compounds while on the basis of the detection frequency method only 36 of these possessed an odor activity in the MPFs. The developed odorous compounds varied according to the starting sample and depending on the degree of hydrolysis. DH was an important index in the preparation of meat flavors; in particular, compounds with a DH range of 25.92%–30.89% produced a wider range of odor-active compounds through thermal reaction.

GC-O analysis was also used to discriminate between fresh and frozen lamb meat in a study attempting not only to evaluate the chemical basis of the aroma of grilled lamb but also to determine the major aroma changes linked to meat freezing [166]. Experiments were performed using a multidimensional gas chromatography–olfactometry–mass spectrometry (GC × GC-O/MS). The experimental design included the GC-O analysis of nine different extracts: two blanks, two samples of fresh grilled meat, two of fresh grilled muscle, two of previously frozen grilled meat and one of fresh grilled fat. The main advantage of this methodology compared with others presented in recent years [167] is that it was performed *in vivo* with the help of volunteers; therefore, the relevant processes occurring inside the mouth such as chewing and enzyme action from saliva were taken into account. This work was able to detect the most important compounds in the aroma of lamb during grilling and consumption and for the first time 2-isopropyl-3-methoxypyrazine, 2-methylbenzaldehyde and vanillin were detected in lamb.

GC-O with statistical analysis was applied to aromatic caramel which is widely used in the food industry especially as a food flavoring. Caramel can be liquid or solid and brown to dark brown. It is soluble in water and obtained by the controlled action of heat on sugars [168,169]. Products resulting from the thermal degradation of sugars, like coffee, and odorant properties are closely linked to the volatile fraction which represent 5%–10% of their total mass; GC-O and GC-MS could be used to identify and structurally characterize those compounds. The relationship between physicochemical and sensory data sets was studied by means of multivariate statistical tools such as Partial Least Square (PLS) regression. The odorant compounds detected belonged to several chemical classes: oxygenated heterocycles, carbocyclic compounds, carboxylic acids, phenolic compounds, esters, aldehydes and carbonyled compounds. Among them, 13 odor zones were related to oxygenated heterocycles, carbocyclic compounds and carboxylic acids. These results suggested the importance of these three chemical classes to the odorant properties of burnt sugars.

Extensive reviews dealing with the application of GC-O on dairy products, including tables of classified odorants according to their chemical classes are present in literature [25,170]. In one study, the change in aroma composition of cow's milk during heating and fermentation was investigated [19]. Seven common odorants were found in four different types of raw milk: dimethylsulfone, ethyl butanoate, ethyl hexanoate, heptanal, indole, nonanal, and 1-octen-3-ol. Heating brought about the formation of four common odor-potent compounds: hexanal, 2-nonanone, benzothiazole, and δ-decalactone. Instead, fermentation resulted in the formation of 1-octen-3-one, methional, 3-methylbutanal, and butyric acid.

#### 3.1.3. GC-O as a Discrimination Tool among a Family of Foodstuffs

Discriminant analysis between foods and among varieties of the same class of foods is another of the applications for GC-O. Several studies have used GC-O to verify odorant compounds in coffee and to compare the highly volatile odorants of the powders and brews prepared from roasted Arabica and Robusta coffees. A brew was also obtained from a soluble coffee powder to detect odorants with a boiling point lower than that of the extraction solvent [171]. Furthermore, GC-O was successfully used for the aroma analysis of coffee flavor by evaluating defects in green coffee beans due to microorganisms responsible for the formation of off-flavors [172]. In this study, the authors compared green Arabica coffee beans from Mexico, obtained by a dry post-harvest treatment, with a coffee bean of identical origin having no noticeable organoleptic defect and then a moldy/earthy character defined. A frequency detection method was applied for obtaining the olfactogram. GC sniffing profiles made it possible to locate zones with a typical moldy/earthy character and to attribute moldy/earthy off-flavor to six substances.

In another study, GC × GC-O was extensively used to check odor compounds in complex samples such as brewed coffee. This led to the detection of numerous odor compounds which could be well resolved and identified in both roasted and brewed coffee [28]. The authors clearly demonstrated that certain odor regions correspond to several overlapping compounds in 1D GC, however, these co-eluting peaks were well resolved in the 2D axis of GC × GC analysis. Moreover, it was found that a short 15 m 1D column could exacerbate the resolution problem in 1D analysis and a better resolution could be obtained on a longer column. However, the latter would add time and aggravate the problems of assessor's fatigue when an olfactometric port is used.

**Figure 6.**
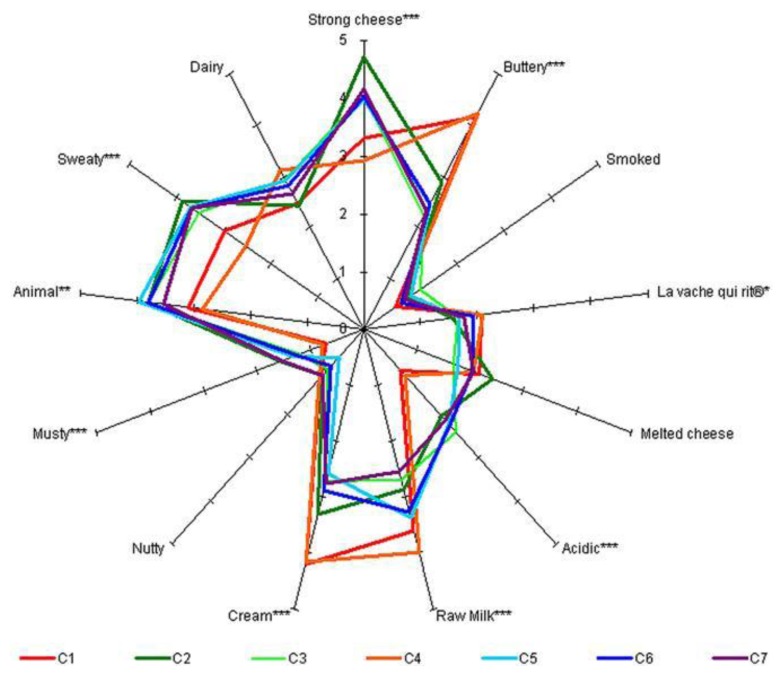
Mean ratings of the 13 odor attributes for the seven (C1–C7) semi-hard cheeses (13 judges; 3 repetitions). Significant differences are shown: * significant at *p* < 5%; ** significant at *p* < 1%; *** significant at *p* < 0.1% (reprinted from [173] with permission from Elsevier).

An interesting study has recently been carried out [173] on 7 different semi-hard French cheeses. The work investigated the relationship between physicochemical and sensory data sets using multivariate statistical tools. It began by identifying thirteen odor attributes. A characterization was made using a conventional AFNOR sensory profile to correlate the perceived orthonasal aroma compounds with those extracted from the headspace and analyzed with GC-O [174,175]. Only 9 of the 13 odor attributes showed any significant difference among the seven cheeses (analyzed with ANOVA). As illustrated in Figure 6, the “Smoked”, “Dairy”, “Nutty” and “Melted Cheese” attributes did not have enough discriminant power to differentiate the 7 cheeses.

**Figure 7.**
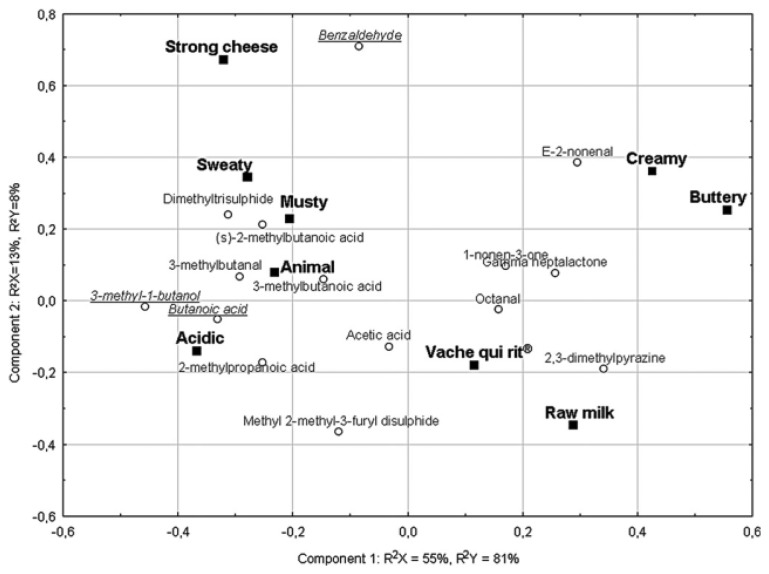
Bi-plot of the two first components as a result of PLS analysis of the sensory profiles (Y matrix, black) and the GC-O intensity measurements for the odor-active compounds (X matrix, grey) (reprinted from [173] with permission from Elsevier).

In the second phase, the odor-active compounds were detected using 8-way gas chromatography-olfactometry (GC-O/8). The single-port GC-MS/O chromatogram and the GC-O/8 aromagrams were aligned, by means of standards, and processed with AcquiSniff software [26,173,176]. It was found that some odor zones were present in all of the cheeses while some were specific to only one or a few cheeses. ANOVA was carried out on the intensity ratings from the eight judges for each odor-active compound and it was noted that a total of 15 compounds significantly discriminated the cheeses. Evaluation of the correlations between the sensory profile and GC-O was performed using PLS with a nonlinear iterative partial least squares (NIPALS) algorithm to explain sensory ratings from the GC-O intensities. The total intensity of GC-O data was considered as the X matrix and the mean sensory scores as the Y matrix. The optimal number of dimensions for model prediction was determined by cross-validation. The results of the bi-plot correlation between the sensory profile and the GC-O data are reported in Figure 7. The sensory attributes, “Buttery”, “Cream”, “La vache qui rit” (cheese type), and “Raw Milk”, were correlated with odorant compounds having green (*E*-2-nonenal and 1-nonen-3-one) and roasted (2,3-dimethylpyrazine) characteristics. The attributes, “Musty”, “Animal”, “Sweaty” and “Acidic”, were mostly correlated with odorant compounds having cheesy, sulphury, rancid and chocolate characteristics as butanoic acid and 3-methyl-1-butanol.

The work underlines that the use of a simultaneous multi-sniffing detection system aims to overcome a well-known drawback of the GC-O technique, that is the lack of repeatability and reliability of some measurements with respect to instrumental ones, especially when only one assessor is employed.

### 3.2. Fragrance Applications

Chemical characterization methods coupled with olfactometric detection can be a powerful tool for the sensorial characterization of odor. In the field of fragrances, this method can be used for research purposes or for improving industrial manufacturing processes. The use of fragrance materials dates back to antiquity, when spices and resins from animal and plant sources were used in perfumery. Today, perfumers work with several thousand natural or synthetically manufactured ingredients to create different fragrance compositions [177]. The combination of commercial demands with the development of monodimensional gas chromatography (GC), GC/mass spectrometry (GC/MS), and GC-olfactometry (GC-O) has produced an explosive acceleration of the evolution of flavor and fragrance materials [29]. Essential oils, extracted from the source array, or perfumes used as such, are subject to quality control as well as chemical and odorous characterization in industry where the main goal is to make these products as pleasant as possible and to confer a characteristic odor. Hence, GC-O can be readily used because of its ability to efficiently separate and characterize the principal molecules constituting these matrix such as terpenes, aldehydes and alcohols.

**Figure 8.**
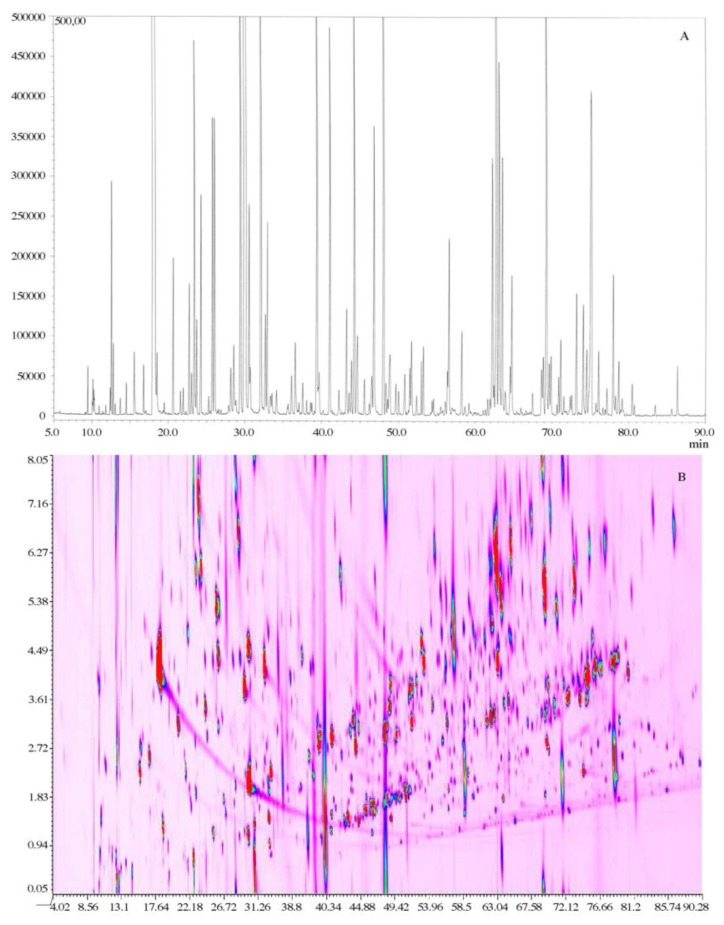
GC-O chromatogram (**A**) and GC × GC-O 2D plot (**B**) of a commercial perfume achieved without (**A**) and with (**B**) cryogenic modulation (reprinted from [29] with permission from Elsevier).

For fragrance recognition, innovative FFNSC (flavor and fragrance natural and synthetic compounds) libraries are used; the best matche can be found between an investigated compound and the target one based on similarities in fragmentation and the closeness of the RI values [25,178]. In the case of particularly complex mixtures or when compounds are present at trace-level concentrations, multidimensional gas chromatography techniques (GC × GC) are preferred in order to obtain a better separation of the different compounds. The main advantage of using GC × GC-O is shown in Figure 8. Indeed, the comparison of a GC-O chromatogram and a GC × GC-O 2D plot of a commercial perfume has revealed that these type of matrixes are very complex and that conventional GC-O analyses cannot adequately record the presence of all constituents [29].

Using GC-O analysis, detailed reports of the chemical and aroma components of some essential oils have been compiled. For example, GC-O analyses revealed several compounds related to the camphoraceous, herbaceous and fresh odor that characterizes the essential oil of *Tarchonanthus camphoratus* L. It is notable that the majority of the compounds having the highest intensity score belonged to the class of oxygenated monoterpenes which are considered the most expressive class of terpenes used in perfumery [179].

While studying *P. mirifica*, a commercially available Thai leguminosae plant considered to be a rejuvenating drug, α-necrodol, a terpene having anti-insect activity in the *Pueraria* genus, was detected for the first time by Yagi *et al.* [57,180,181]. Moreover, odor analysis allowed to distinguish green odor (C9 aldehydes group, such as phenylacetaldehyde and (2*E*)-nonenal) and sweet odor (monoterpene alcohols, such as geraniol). Hence, the main advantage of GC-O analysis lies in the possibility of assigning a characteristic odor to the various compounds present in the same species of plants, in order to obtain information for a possible use of these aromatic samples in food products or in medicinal or cosmetic applications [182].

AEDA analysis has been performed for odor characterization in essential oils. Relevant AEDA results were found in the GC-O characterization of *Scutellaria laeteviolacea* essential oil: relative flavor activity (RFA) was calculated using the equation reported by Song *et al.*, starting with the FD-factor:

(4) $$\text{RFA}=\log\frac{\text{FDfactor}}{S^{0,5}}$$

In the formula reported above, the FD-factor is the dilution factor while S is the weight percentage of the component present in the mixture [183–185]. In GC and GC-MS analyses, 100 compounds were characterized and seventeen peaks were confirmed by sniffing with GC-O. The work demostrated that the compound germacrene D and *Scutellaria laeteviolacea* essential oil had the most similar smell, even if the latter was characterized by a relatively low (0.5) flavor activity and by the highest FD-factor (7). These findings suggest that the relative flavor activity, defined as a new odor unit, and the FD-factor often have no relation to the aroma character of a compound. In other words, even if the RFA of one compound is not comparatively high, it often contributes significantly to the original odor and can be used when considering flavor activity [55].

*Clinopodium tomentosum* (Kunth) Govaerts essential oil was studied with GC-O in order to calculate threshold odor concentration (TOC) values of the main odorants in a mixture [53]. As displayed in Table 2, Benzo *et al.* found a good agreement between the calculated and measured TOC values of a few odorants in the essential oil. They established a relationship between the odorant concentration at the sniffing port and that in the injected solution using the TOC value of limonene as a reference compound.

### 3.3. Environmental Odor Applications

Odors produced by anthropic sources are a complex issue because they directly affect both the environment and the human quality of life. Industrial plants and farms are often a source of bad odor, hence, their close proximity to residential zones can lead to complaints by local residents [4,186,187]. Furthermore, odors can strongly affect people's daily life and wellbeing since they may provoke both physiological symptoms (respiratory problems, nausea, headaches) and psychological stress [188,189].

The official methodology for odor emissions assessment is dynamic olfactometry, a sensorial technique standardized by international technical laws [190,191]. It is based on the use of a dilution instrument, called an olfactometer. This device releases the odor sample diluted with odor-free air at precise ratios to a panel of human assessors who have been selected according to their perception threshold for a reference gas. The odor concentration, usually expressed in odor units (ou/m^3^), is numerically equal to the dilution factor required to reach an odor threshold, which is the minimum concentration perceived by 50% of the population [190,191]. However, it is not sufficient to completely evaluate a case of olfactory nuisances for various reasons [192]:

- Continuous and field measurements, which are useful for monitoring industrial processes causing odor emissions, cannot be performed;
- Odor concentration refers to the whole odor sample, without discriminating between single chemical compounds and their contribution to that concentration;
- Odor samples require rapid analysis since they are instable and difficult to store;
- Olfactometry can be quite time-consuming and expensive, and the frequency and duration of the analyses are limited.

In order to overcome these limitations, sensoristic and analytical methodologies as well as others are widely employed and their information are often integrated to achieve a more complete understanding of olfactory nuisance cases. The use of a hybrid instrumentation such as GC-O has been shown to provide interesting information in the environmental field due to the coupling of the chemical characterization with sensorial perception related to the single compounds eluted by the column.

A considerable number of scientific works dealing with the use of GC-O have focused on odor assessment produced by different types of animal farms. The activities connected with high density livestock operations can produce several hundred volatile organic compounds (acids, alcohols, aldehydes, amines, volatile fatty acids, hydrocarbons, ketones, indoles, phenols, nitrogen and sulfur compounds) yet relatively few of them are responsible for the typical odor of these environments. Hence, the aim is to the extract compounds that are actually responsible for the primary odor impacting livestock environments. Several factors can complicate this task; for instance, the variability among species, manure management systems and animal production practices [193].

**Figure 9.**
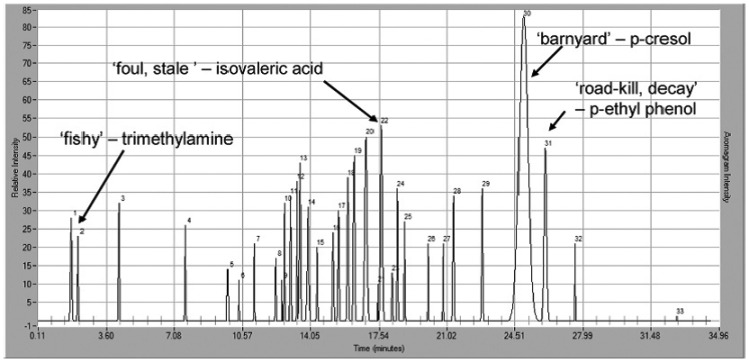
Aromagram for 4 h SPME fiber collection 20 m downwind (“near” site) from commercial beef cattle feed yard (reprinted from [193] with permission from Elsevier).

**Figure 10.**
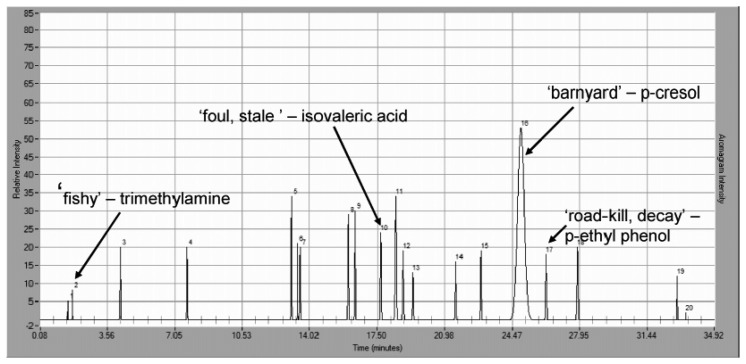
Aromagram for 4 h SPME fiber collection 2,000 m downwind (“distant” site) from commercial beef cattle feed yard (reprinted from [193] with permission from Elsevier).

Using GC-O to profile odor is a functional approach for defining, prioritizing and tracking livestock odorants. Wright *et al.* [193] have studied the odor profiles produced by swine and beef cattle operations by optimizing the collection time and/or increasing the distance from the odor source. The increased distance showed a significant reduction in the total number of detectable odors, as shown in Figures 9, 10. The authors found that p-cresol was the only significant olfactory response even 2,000 m away from the odor site, meaning that it could be considered as a surrogate parameter correlated to odor.

A lot of studies have reported that most of the odor produced by swine barns is carried on dust [194–196] and that its reduction is possible using dust filters for Particulate Matter (PM) [197,198]. To further understand this aspect, GC-MS/O was used to identify odorous VOCs adsorbed/absorbed on different size swine barn dust (PM_1_, PM_2,5_, PM_10_ and TSP). The study of the aromagrams of the VOCs extracted from TSP filters at different SPME sampling times allowed Cai *et al.* [81] to identify the key odorants for the different granulometric fractions of the particulate matter as well as identify what is actually carrying the odorous compounds.

**Figure 11.**
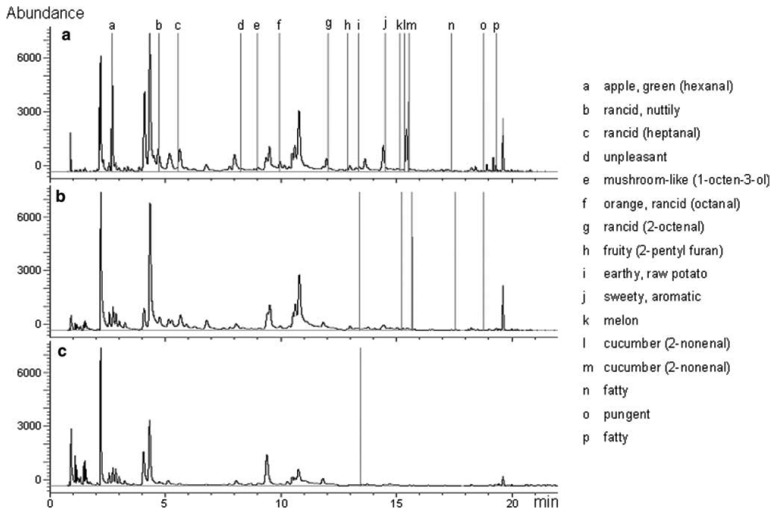
FID/O-chromatograms of waste gas from a fat refinery obtained prior to and after waste gas treatment: (**a**) untreated waste gas; (**b**) after bioscrubber; (**c**) after biofilter; a–p odor signals (reprinted from [80] with permission from Elsevier).

GC-MS/O is also widely employed for determining the efficiency of different treatment systems for odor emission reduction and for developing a suitable and cost effective strategy for implementation. In particular, the sensorial data associated to each single compound are able to indicate what compounds, among those that really contribute to odor, have to be mitigated in order to reduce odor emissions. This aspect has a practical implication in the development of opportune abatement systems specific for the compounds causing odor emissions [78,83]. For example, GC-MS/O was used to evaluate the effectiveness of topical zeolite applications to mitigate VOCs and odor from simulated poultry manure storage [199]. It was also used to characterize the odorants either before and after the application of abatement products (activated carbon, silica gel and zeolite) in order to reduce odor compounds emitted by broiler litter materials [200] or prior to and after waste gas treatment from a fat refinery [80], as shown in Figure 11. An analogous approach was used by Chen *et al.* [78] for examining two types of wood chip-based biofilters as common abatement systems.

In the same way, Agus *et al.* [74,201] set up a noteworthy application of GC-O in environmental matrixes. Their study identified trace amounts of odorous organic compounds in drinking water that was obtained from highly treated wastewater during the different phases of the treatment.

Some authors have tried to combine the GC-O results with those acquired from other approaches. The goal of this integration may be to discover useful correlations in order to obtain a better understanding of a case study or to test the effectiveness of GC-O in determining the principal odorants in a mixture. The work of Sohn *et al.* [202] can exemplify the former objective. Their study combines GC-O with a real time monitoring system (an artificial olfaction system) to measure in shed odor concentrations at two different poultry farms. The results were combined with ventilation rates and weather data to calculate the odor emission rate (OER) throughout the batches, observing that OERs varied significantly between farms. This variation was linked to changes in ventilation rates, bird activity and other management and environmental factors. Similarly, GC-MS/O results showed different chemical profiles during the poultry production cycle. The matrix was first dominated by terpenes originating from the bedding material of young birds while later it became populated by aldehydes, ketones and sulphides as the bedding became soiled with manure.

In other studies, the results of GC-O were integrated with the odor concentrations measured by dynamic olfactometry. In these cases, correlations between the concentrations of odorous VOCs and measured odor units were investigated in order to define the main compounds contributing to the entire perception of odorous VOCs [83,84]. In another case, Zhang *et al.* [87] characterized the odor emissions produced at swine and dairy sites and associated odor intensity and hedonic tone to compounds. They were also able to demonstrate that concentrations of odorous compounds correlated well with the measured log stimulus intensity.

For the purpose of demonstrating the effectiveness of GC-O in identifying the principal odorants in a mixture, Trabue *et al.* [86] compared the results of GC-O with odor activity values (OAV) obtained in open cattle feedlots. Here, the sampling was carried out at the source and far from it. Based on OAVs, the chemical characterization of the source revealed that Volatile Fatty Acids (VFA) were the principal contributors to odor, followed by phenols and indoles. At 250 m downwind from the source, the total OAV declined by almost 95% while at 3.2 km downwind it decreased to a value of less than one, meaning that no odor should be present, unlike to what was described. On the other hand, GC-O results revealed that, at 250 m downwind of the feedlot, a panelist could not perceive pentanoic acid odor, despite its concentration was above its OT while perceived 4-ethylphenol with an OAV less than one. These results indicate that an approach based solely on OAVs is not sufficient to describe odor characteristics because of the OT value uncertainty, as revealed in literature.

### 3.4. Material Applications

**Figure 12.**
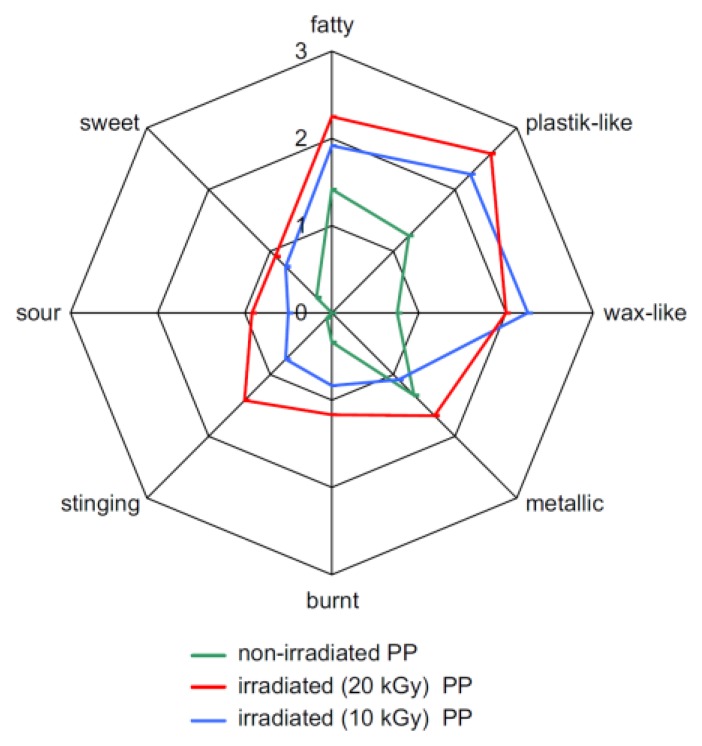
Orthonasal comparative flavor profile analysis (cFPA) of three powdered PP samples. The data are displayed as mean numerical values of the sensory evaluations (three sessions with six panelists each) (Reprinted from [52] with permission from Elsevier).

The GC/MS-O method has proven to be a useful and reliable tool for the detection and identification of odor active VOCs responsible for off-flavors coming from a wide range of materials. Material applications have two main goals. One is to inform industry of manufacturing processes and any possible improvements in that practice. The other is to find replacements for raw materials that generate odorous compounds with other odorless compounds in order to avoid or, at least, reduce odor nuisance. This research is important for food packaging materials since it can search for ways of avoiding the possible migration of materials into food or other susceptible products where they may cause unexpected and unsightly changes. Tyapkova *et al.* used the GC-O approach to characterize the volatile chemicals that forms in sterilization processes, with 60Co γ-irradiation in the presence of oxygen, of polypropylene (PP) packaging materials, used in food, pharmaceutical or cosmetic fields [52]. The authors compared VOCs emission from irradiated (rays at 10 and 20 kGy) and not irradiated polypropylene, including a sensory evaluation with a panelist. Besides compositional changes in volatile odorous substances from PP during treatment, the results showed a shift towards a different odor descriptor (fatty, sweet, sour, burnt, stinging, metallic, wax-like, plastic) depending on the γ-irradiation condition. The experimental results are reported in Figure 12 as orthonasal comparative Flavor Profile Analyses (cFPA).

Nowadays, GC-O methodology is widely applied to evaluate VOCs and odor emissions from waste-recycled innovative materials, employed both in the automotive industry and for construction, furniture and consumer products. Although the recycling of waste materials is well established and widely applied, it has to satisfy chemical requirements in order to obtain safety certification.

Felix *et al.* applied HS-SPME extraction and GC-O/MS analysis to wood-plastic composites (WPC) produced with landfill derived plastic and sawdust in order to characterize VOC emissions and to assess the impact of the odor on the end-users [82]. This study found that the WPC prototype had a characteristic odor profile and that many of the compounds observed are related to reprocessed materials, confirming the hypothesis that repeated recycling can generate thermo-oxidative degradation (Table 3). In addition to the findings reported by a previous study [203], Felix *et al.* stressed that the degradation of a polymeric matrix produces a characteristic odor associated with aldehydes, ketones and carboxylic acids, whereas the degradation of the lignocellulosic component releases acetic acid, formaldehyde, formic acids, aldehydes and other acids [82].

The applied combination of sensory assessment and GC/MS analysis also seems to be a useful approach in the effort to eliminate unwanted odors from building products. In this regard, Knudsen *et al.* focused attention on VOCs and odor emissions derived from environmentally friendly products with linseed oil (e.g., linoleum, wall paint) that influence the perceived air quality more negatively than similar synthetic products and for a longer period of time [204]. The primary goal of this work was to test if the odor of linseed oil influenced the odor of the final building product. Experimental data obtained indicated that the undesirable odor-active VOCs, having low odor thresholds, had probably originated from the degradation of the linseed oil, due to ozone-oxidation processes. As a result, the authors suggested the use of less odorous linseed oils and gave useful instruction to manufacturers to improve the acceptability of odorous emissions from these building products. Hence, once the objective perception of an odor, for example, by conducting a preliminary olfactometry analysis, is verified, the identification of the principal volatile compounds contributing to the perceived overall odor derived from the object of investigation can be obtained with the application of GC-O/MS.

Another interesting application of GC-O/MS investigation has evidenced the link between the physical and chemical properties of oak wood with the chemical composition, olfactory and gustatory qualities of wines fermented and/or aged in oak barrels. The aim of a study carried out by Díaz-Maroto *et al.* was to investigate the sensory importance of oak wood VOCs in order to evaluate the contribution of wood-derived volatile compounds to the overall aroma of oak-aged wines [51]. This study demonstrated that oak wood treatments such as seasoning and toasting, as well as other factors like tree species and geographic location, can modify both the physical and chemical qualities of the wood, resulting in the characteristic aromas (fruity, fresh/green/grassy and floral) of wines. Moreover, trans-2-nonenal and decanal which can transmit unpleasant aromas to wine were detected in non-toasted oak woods suggesting that the toasting treatment of the wood could reduce the problem.

Taking into account the aforementioned applications of GC-O/MS methodology for the optimization of production processes and the improvement of the quality and odor acceptability of a final product, it can be stated that GC-O/MS methodology has revealed potentials not associated to other analytical techniques.

### 3.5. Medical Applications

One of the more recent and promising applications of GC-O is in medical research where the study of volatile and odorous profiles of biological matrixes is mainly used to aid in the diagnoses of diseases and dysfunctions. In this area, interesting results have been obtained from the characterization of volatile and odorous profiles in human urine. Indeed, the study of urine could yield a wealth of physiological information and increase the understanding of metabolization and excretion processes of low molecular weight compounds originating from dietary or endogenous sources. Moreover, changes in individual profiles can be potential indicators and mechanistic clues of deviations or even misbalances in physiological conditions induced by diseases or hormonal changes. However, since the application of modern analytical tools in volatile analysis in urine has been limited, the diagnostic potential of urinary volatile fraction is not yet fully understood.

Recently, Wagenstaller and Buettner [49] have applied GC-O methods to evaluate a combination of comprehensive chemo-analytical and human-sensory methods for the characterization of human urine odorants. A total of 14 odorants were detected in most of the untreated urine samples and 24 odorants in the glucuronidase-treated samples. Most of these potent odor compounds had not previously been detected in human urine when traditional methodologies were used. The study highlighted the high analytical potential of the combined approach in elucidating the fate of volatile/odorous food constituents or substances originating from other origins (e.g., pharmaceuticals) and in characterizing the excretion of endogenous substances. This approach may very well pave the way for a better understanding of the diagnostic potentials of the odorous and volatile fraction in human urine.

GC-O was also used to study the influence of thermal reaction and microbial transformation on the odor of human urine after being boiled or fermented [72]. The headspace of the samples was analyzed to isolate and identify the malodor-generating bacteria present in the urine of healthy people. The study showed that urine composition may certainly influence pH, bacterial composition and urine odors. Incubation of freshly collected urine revealed that the maximum concentration of total bacterial counts was reached after 4 days at room temperature (20–25 °C) and after 1 day at 37 °C. Indeed, most of the volatile compounds were generated during the first hours of incubation. Analytical comparisons between boiled and fermented urine revealed that the incubation of sterile urine with a bacterial mixture of *E. fergusonii*, *Enterococcus faecalis*, *Citrobacter koseri*, *S. agalactiae* and *M. morganii* produced a characteristic aged urine odor.

Shirasu *et al.* identified dimethyl trisulfide (DMTS) as the main odorant responsible for severe malodors in some advanced cancer patients by performing gas chromatography-mass spectrometry-olfactometry analysis of volatiles from fungating cancer wounds [205]. The intensity and quality of the body odors emitted from the fungating wounds of three female patients with breast cancer and of two male patients with head and neck cancer were examined. In particular, sterile gauze pads were placed on the wounds for 6–12 h and headspace volatiles were extracted using SPME fiber and then analyzed with GC-O. The study produced useful data for the development of a strategy to prevent or reduce the DMTS odor which helped to improve the quality of life of the patients.

In another study, the strong “maple-syrup” odor which accompanies fenugreek ingestion was investigated by Mebazaa *et al.* [206]. The odor active compounds present in HS-SPME armpit sweat extracts collected before and during fenugreek ingestion periods were analyzed with GC-O and evaluated by a panel of eight experienced assessors. Collected data were treated using the frequency of detection methodology. Among the 44 compounds identified, 10 were detected by assessors before and during fenugreek ingestion such as a-pinene, 6-methyl-5-hepten-2-one, nonanal, 1-octen-3-ol, 2-phenylethyl alcohol and benzenemethanol. Although these compounds had already been identified in human armpit sweat collected from different male and female subjects, this is the first study of their effect in the overall odor of human armpit sweat.

## 4. Conclusions

In recent years, researchers involved in the study of odorous substances have recognized the great potential of gas-chromatography/olfactometry in their work as it can simultaneously provide analytical and sensorial information about an odor mixture. However, the complexity of this matter has limited the application of GC-O whose first application dates back to 1964. The many factors linked with chromatographic parameters as well as the variables related to human perception and panel assessment represent a challenge for researchers. The goal is to find the best use of these components in order to improve both the practical experimental use of GC-O and the quality of the data obtained.

GC-O has been extensively employed in food aromas and fragrance studies. This application has been primarily industrial and driven by commercial desires to improve of the quality of products in order to make them more pleasing and desirable to consumers. In the recent years, researchers have also begun exploring GC-O applications in environmental, material and medical areas. One remarkable application of GC-O is in the medical field where the characterization of volatile and odorous profiles of biological matrixes could be useful for carrying out screening analyses of human diseases and dysfunctions.

GC-O is becoming a reliable and reproducible method for characterizing the odor footprint of complex mixtures of chemicals of diverse origin. Noteworthy application improvements include the introduction of new and efficient interfaces between the GC and olfactometric port, the reduction of subjective error perception and anosmia by increasing the number of simultaneous panelists (up to 8), and the use of bi-dimensional GC techniques and sophisticated statistical programs for data processing and interpretation. In conclusion, GC-O may be considered one of the most valuable methods in odor research today. Future improvements in the effectiveness of GC-O application should include a standardization of the different approaches being used. This would be valuable in order to estimate the sensory contribution of a single odor active compound in a complex mixture as well as in the resulting aromagrams.

## Figures and Tables

**Table 1.** Odor-active compounds in yerba mate detected by GC–O with MF ≥ 50 (reprinted from [69] with permission from Elsevier).

**LRI^a^**	**%MF^b^**	**Descriptor**	**Compound**
1154	53	Herbaceous, sweet	Myrcene
1278	76	Citrus	Octanal
1294	53	Mushroom	1-Octen-3-one
1340	74	Moss	6-Methyl-5-hepten-2-one
1450	68	Flower	(*Z*)-linalool oxide (furanoid)
1478	76	Nuts	(*E,Z*)-2,4-heptadienal
1490	68	Nuts	(*E,E*)-2,4-heptadienal
1510	84	Mushroom	(*E,Z*)-3,5-octadien-2-one
1528	66	Mushroom	(*E,E*)-3,5-octadien-2-one
1540	65	Flower	Linalool
1745	61	Flower	Geranial
1798	76	Sweet	Nerol
1832	71	Apple	β-Damascenone
1843	76	Flower	α-Ionone
1936	79	Sweet	β-Ionone
1987	53	Oxidized, metallic	(*E*)-4,5-epoxy-(*E*)-2-decenal

a LRI in Carbowax™;

b MF, modified frequency.

**Table 2.** TOC values calculated using AEDA method in GC-O technique and measured by dynamic dilution olfactometry (reprinted from [53] with permission from Elsevier).

**No.**	**Compound**	**Calculated TOC (μg/m^3^)**	**Measured TOC (μg/m^3^)**	**Descriptor**
1	1-Octen-3-ol + 6-methyl-5-hepten-3one	0.223	2.36 and 18.89	Mushrooms
2	1,8-Cineole	2.67	5.08	Balsamic
3	Isomenthone	40.451	n.d.	Wine bottle stopper
4	Isopulegone	0.076	n.d.	Minty
5	Pulegone	0.884	1.87	Minty
6	*cis*-Piperitone oxide	22.427	n.d.	Minty

**Table 3.** Olfactory description, chemical identity and modified frequency percentage MF (%) for each odorant identified in WPC prototype (Adapted from [82] with permission from Elsevier).

**RT (min)**	**KIexp ^a^**	**KIref^b^**	**Odor Descriptor**	**MF (%)**	**Compound**
7.268	1011	970	Diacetyl, cream, sweet, yogurt, curd, wood, fruity	83	Diacetyl (2,3-Butanedione)
9.188	1120	1084	Grass, herb, green, flower, solvent, chemical	76	Hexanal
10.468	1177	1150	Fruity, ester, candies, jelly, plastic, varnish	53	m-Xylene
13.849	1321	1280	Aldehyde, medicine, chemical, herb, flower, field, lemon, grapefruit, orange	82	Octanal
16.314	1424	1385	Aldehyde, bleach or lemon cleaner, unpleasant	77	Nonanal
17.668	1481	1450	Acid, unpleasant, solvent, glue, sweat, sunflower seeds	79	Acetic acid
18.732	1527	1484	Aldehyde, powdered sugar, acid	51	Decanal
19.029	1540	1490	Gas, burnt, green shield bug, fresh wood, fried, oily	71	Acetylfuran
19.316	1553	1491	Dry fruit, nut, almond, mold, dense	58	Camphor
21.655	1658	-	Cheese, rancid cheese, butiric or propanoic acid	77	Unknown
22.611	1702	-	Acid, cheese, butiric acid	76	Unknown
23.080	1724	1720	Bug, nail polish remover, naphthalene balls	53	α-Terpineol
26.123	1872	1829	Acid, trash, waste, foot, wood, hair removal wax, licorice	74	Hexanoic acid (caproic acid)
26.508	1891	1859	Phenol, shoeshine, medicine, sweat, bug, vanilla	65	2-Methoxyphenol (Guaiacol)
28.105	1973	-	Phenol, aromatic, sweet, zinc oxide adhesive plaster, opium, hospital	74	Unknown
30.857	2075	-	Unpleasant, acid, wood, manure	65	Terpin hydrate
32.727	2130	2198	Lactone, burnt, car tire, rubber	35 ^c^	2-Methoxy-4-vinylphenol (4-Vinylguaiacol)
35.655	2204	2358 ^d^	Flower, salt water, hair removal wax	38 ^c^	Diethyl phthalate (DEP)
39.374	2270	2569	Car tire, vanilla, soluble chocolate powder, burnt	53	Vanillin

a Kovats retention index calculated from BP-20, 30 m column;

b Kovats retention index reported in the Flavornet Database (Carbowax™ 20 m column);

c Compounds with MF < 50% but relevant to the sample;

d Kovats retention index calculated from DB-Wax 60 m column.
